# A liver DNA synthesis promoter induced in rat plasma by injection of dimethylnitrosamine (DMNA) or thioacetamide.

**DOI:** 10.1038/bjc.1987.122

**Published:** 1987-06

**Authors:** J. J. Díaz-Gil, G. Sánchez, L. Santamaría, C. Trilla, P. Esteban, P. Escartín, T. Gea

## Abstract

**Images:**


### A liver DNA synthesis promoter induced in rat plasma by injection of dimethylnitrosamine (DMNA) or thioacetamide

J.J. Díaz-Gil[1], G. Sánchez[1], L. Santamaría[2], C. Trilla[1], P. Esteban[1], P. Escartín[3] & T. Gea[4]

[1]*Department of Experimental Biochemistry, Clínica Puerta de Hierro;* [2]*Department of Morphology, School of Medicine, Universidad Autónoma;* [3]*Department of Gastroenterology, Clínica Puerta de Hierro; and* [4]*Department of Clinical Biochemistry, Clinica Puerta de Hierro, 28035 Madrid, Spain.*

**Summary** The appearance of a liver DNA synthesis promoter (HP) in rat plasma after dimethylnitrosamine (DMNA) or thioacetamide injection was investigated. After 48 h, DMNA ($30\,mg\,kg^{-1}$ body weight) produced liver (centrilobular) necrosis and intense hepatic regeneration, as assessed by microscopic observations of liver slices, as well as augmented transaminase levels; HP was detectable under these conditions. After 5 days, transaminases and HP returned to normal values (the latter undetectable), coinciding with a lack of necrotic zones. At 60 mg DMNA $kg^{-1}$ body weight, necrotic areas were more marked and transaminases and HP levels higher after 48 h than with the lower dose; these increases were even more pronounced at 90 mg DMNA $kg^{-1}$ body weight.

After thioacetamide injection ($200\,mg\,kg^{-1}$ body wt) the situation at 48 h was very similar, with focal, centrilobular necrosis, frequent regenerative signs, high transaminases and detectable HP. Rats recovered after 7 days in a similar fashion as with DMNA. At 400 mg thioacetamide $kg^{-1}$ body weight, necrotic areas and regeneration zones were more widespread and transaminases and HP higher after 48 h than with the lower dose.

On account of the differing modes of action of DMNA and thioacetamide in rat liver, it is proposed that the appearance of HP activity in plasma could be related to the regenerative process that follows hepatotoxic damage.

Although the steps that follow a chemical assault on the liver are very poorly understood, hepatic recovery, in terms of functionality and disappearance of necrosis after several days of injection of various kinds of aggressive agents, is a well-known phenomenon (Leevy *et al.*, 1959; Ivanetich *et al.*, 1984). This apparent recovery has been postulated to be preceded by a regenerative process, but at present, evidence determining the presence and nature of regenerating factor(s) is scanty, although a DNA-synthesis promoter activity was detected in plasma after thioacetamide administration (Morley & Boyer, 1977).

Our group has recently reported (Díaz-Gil *et al.*, 1986*a*) the purification of a liver DNA synthesis promoter (HP) from plasma of partially hepatectomized rats. The HP preparation shows a single band on SDS-polyacrylamide gel electrophoresis (silver-stained), with a molecular weight of 64,000, and appears to be a protein. When injected into mice (150 ng HP/mouse), an increase in liver DNA synthesis is detected. At the same dose, HP increases the mitotic index (MI) of mouse hepatocytes. Its action is organ-specific (acting on liver, but not on spleen, kidney, lung or brain). In primary liver cultures, 1–10 ng HP $ml^{-1}$ produce an increase of [$^3$H]thymidine uptake by DNA. In this *in vitro* system, it increases the uptake of $^{22}Na^+$ immediately after addition.

Herein we detect HP in plasma of rats injected with dimethylnitrosamine (DMNA) or thioacetamide. Maximal concentration of HP in plasma seems to coincide with higher levels of transaminases and more widespread necrotic areas in the liver, while it proves undetectable in plasma when transaminases return to normal values and necrosis disappears.

The possible importance of HP in hepatic regeneration has been reinforced by the recent publication by our group of a similar activity, present in plasma of humans with acute viral hepatitis (Díaz Gil *et al.*, 1986*b*).

### Materials and methods

### *Reagents*

Chemicals were purchased from Sigma, Bio-Rad, Merck and Pharmacia, and [$^3$H]thymidine ($20\,Ci\,mmol^{-1}$) from New England Nuclear.

### *Animals*

Wistar rats (90–110 g body weight) were subjected to either DMNA or thioacetamide injection experiments, or to partial (70%) hepatectomy following the method of Higgins and Anderson (1931). Rats were injected i.p. with either DMNA or thioacetamide (using saline as vehicle) at various doses, and were sacrificed at times indicated in the **Results.** Heparinized blood was collected by cardiac puncture and plasma was obtained after centrifugation. The HP was purified from plasma in every case, whether from DMNA or thioacetamide-injected, normal or partially hepatectomized rats, using the isolation procedure published by our group (Díaz-Gil *et al.*, 1986*a*). In brief, it consists of three main chromatographic steps: Sephadex G-75, DEAE-cellulose and hydroxylapatite. The final fraction shows a single band in SDS-polyacrylamide gel electrophoresis, exhibiting activity as a DNA synthesis promoter either *in vivo* or *in vitro*.

The identity of molecular mass of the HP preparations in the different cases was checked by SDS-polyacrylamide gel electrophoresis following the method of Laemmli (1980). Coomassie blue was used as dye.

The activity of the HP preparations was monitored by i.p. injection into Swiss mice (25–29 g body weight). After 24 h, $1\,\mu Ci\,g^{-1}$ body weight of [$^3$H]thymidine was injected into each animal and 1 h later, mice were sacrificed by cervical dislocation. Liver DNA was extracted by the method of McManus *et al.* (1972), measuring total DNA by the method of Burton (1968), and radioactivity by $\beta$-counting using a standard procedure. One unit of activity (UA) is defined as the amount of HP that produces maximum specific incorporation (dpm $\mu g^{-1}$ DNA), nearly 110 dpm $\mu g^{-1}$ DNA following the *in vivo* assay (see Díaz Gil *et al.*, 1986*a* for more details). Due to the peculiar shape of the dose/dependence curve of HP from partially hepatectomized rats, with a maximum at 150 ng HP/mouse, and decreasing activity at lower or higher doses, the analysis of activity requires injection of HP fractions at several doses to ascertain which is the maximal activity dose. (In the first paragraph of the **Results** a practical case is detailed.) The activity of each sample is expressed as UA $mg^{-1}$ protein

Correspondence: J.J. Díaz-Gil.
Received 7 October 1986; and in revised form, 19 December 1987.

injected. Every determination of activity in samples was performed in triplicate. Protein determinations were carried out using the method of Lowry *et al.* (1951).

The concentrations of aspartate aminotransferase (AST) and alanine aminotransferase (ALT) were measured by a Technicon Autoanalyzer following standard procedures. Data are expressed as IU $mg^{-1}$ protein of plasma (mean of three rats). Both enzymes are accepted references for assessing chemically induced liver damage (Alvares, 1982).

For microscopic observations, rats (controls or injected with DMNA or thioacetamide) were sacrificed by decapitation at required times and livers were fixed in 10% buffered formalin (pH 7.2) until use. They were processed by standard histological techniques, embedded in paraffin and cut into 5 $\mu$m sections, stained with hematoxylin-eosin.

### Results

Figure 1A shows changes in the concentrations of ALT and AST in rat plasma after a single i.p. injection of 30 mg DMNA $kg^{-1}$ body weight at different times. Figure 1B shows the concentration of these enzymes after a single injection of 200 mg thioacetamide $kg^{-1}$ body wt. It can be seen that both enzymes reached maximum concentration at 48 h, returning to normal values after 4–5 days (Figure 1A) or 7 days (Figure 1B; in this case, they were checked only at zero, 2 and 7 days). HP activity was detectable in both experiments 48 h after hepatotoxin injection, returning to normal, undetectable values some days later. The indicated values of HP, 1666 UA $mg^{-1}$ of protein (Figure 1A) and 1000 UA $mg^{-1}$ of protein (Figure 1B), signify that it was necessary to inject 600 ng or 1000 ng of HP preparation/mouse, respectively, in each case, to reach the same DNA synthesis stimulation achieved with HP fractions from partially hepatectomized animals (150 ng HP/mouse, with a specific activity of 1/0.00015, equal to 6666 UA $mg^{-1}$ of protein; see **Materials and methods** for more details).

**Figure 1** (a) Enzymatic activities in plasma of rats injected with DMNA, 30 mg $kg^{-1}$ body wt, *vs.* time. *Ordinates*: (left) AST (IU $mg^{-1}$ protein plasma), solid line. ALT (IU $mg^{-1}$ protein plasma), dotted line. (right) HP activity (UA $mg^{-1}$ protein plasma), bars (only 0, 2 and 5-day values are indicated). *Abscissas*: Time (days). (b) Enzymatic activity in plasma of rats injected with thioacetamide, 200 mg $kg^{-1}$ body wt, *vs.* time. *Ordinates and abscissas*: see **Figure 1a**. Only 0, 2 and 7-day values of HP are indicated.

DMNA, at 30 mg $kg^{-1}$ body weight, caused focal peri-centrilobular necrosis with significant periportal regeneration at 48 h (Figures 2B and 2C; compare with control rat liver, Figure 2A)). Liver necrosis was not detectable 5 days after DMNA injection (Figure 2D).

The focal centrilobular necrosis and moderate centrilobular regeneration produced by thioacetamide (200 mg $kg^{-1}$ body wt) in rat liver after 48 h is shown in Figure 3A, necrosis being undetectable after 7 days (Figure 3D).

Figure 4A and Figure 4B show the effects of increasing doses of DMNA and thioacetamide, respectively, in single injections. In both cases, higher doses of the hepatotoxin were paralleled by higher specific activity of HP in plasma, and correspondingly higher concentrations of transaminases, 48 h after injection.

Massive centrilobular necrosis is observed in Figure 2E, where 60 mg DMNA $kg^{-1}$ body wt were injected (to be compared with these aspects as depicted in Figures 2B and 2C).

Accordingly, the centrilobular necrosis is more intense and the centrilobular regeneration more significant at higher doses (compare Figures 3B and 3C, representing 400 mg thioacetamide $kg^{-1}$ body wt, with Figure 3A, representing 200 mg thioacetamide $kg^{-1}$ body wt).

As a quantitative measure of liver regeneration after xenobiotic injection, we counted the number of mitotic figures in several conditions, as indicated in Table I. Mitoses are more abundant in thioacetamide-injected rats at higher doses, being almost absent after 7 days with the lower dose. The time dependence of the number of mitotic figures is also illustrated in the case of DMNA injection. When the dose of DMNA was increased to 60 or 90 mg $kg^{-1}$ body wt, mitoses were not observed.

The concentration of HP in different rats injected with the same dose (either DMNA or thioacetamide) presented some variations, probably due to individual differences in the metabolism of the xenobiotic injected. These individual differences within the same species have been widely

**Table I** Number of mitotic figures in rat liver under several conditions.

Rats were injected with thioacetamide or DMNA, and killed at times indicated. Livers were processed as indicated in **Materials and methods**. One thousand cells were observed in different fields under oil immersion ( × 1000). Figures indicate number in absolute amount and percentage of mitotic figures.

| | *Number of mitotic figures* | *Percentage* |
|---|---|---|
| Control (saline-injected) | ND | ND |
| 200 mg thioacetamide $kg^{-1}$ body wt, after 48 h | 32 | 3.2 |
| 400 mg thioacetamide $kg^{-1}$ body wt, after 48 h | 49 | 4.9 |
| 200 mg thioacetamide $kg^{-1}$ body wt, after 7 days | 1 | 0.1 |
| 30 mg DMNA $kg^{-1}$ body wt, after 48 h | 39 | 3.9 |
| 30 mg DMNA $kg^{-1}$ body wt, after 5 days | 2 | 0.2 |

ND: not detectable.

**Figure 2** (a) Control rat liver. A portal space is observed with no alterations ( × 120) All preparations in **Figures 1 to 4** are hematoxylin-eosin stained. (b) Liver from rat injected with 30 mg DMNA $kg^{-1}$ body weight, 48 h. Intense hepatic regeneration is observed in periportal areas (arrows), with no inflammatory infiltration. Some groups of disperse lymphocytes over the hepatic parenchyma are observed ( × 120). (c) Same conditions as (b). There are clear signs of hepatic regeneration with some mitosis in periportal hepatocytes (asterisk) ( × 1200). (d) Liver from rat injected with 30 mg DMNA $kg^{-1}$ body wt, five days. It is very similar to a control liver with a few signs of periportal hepatic regeneration ( × 120), (c) Liver from rat injected with 60 mg DMNA $kg^{-1}$ body wt, 48 h. Intense hepatic necrosis is observed in centrolobular areas (asterisks) with lymphocytic infiltration ( × 50).

**Figure 3** (a) Liver from rat injected with 200 mg thioacetamide $kg^{-1}$ body wt, 48 h. Portal spaces are well preserved (asterisks); the hepatocytes show numerous regenerative signs around centrilobular veins (star) ( × 120). (b) Liver from rat injected with 400 mg thioacetamide $kg^{-1}$ body wt, 48 h. Intense hepatic regeneration is observed, as well as necrosis and lymphocytic infiltration around centrilobular veins (arrows) ( × 120). (c) Same conditions as (b). Mitotic figures (arrow) and a necrotic hepatocyte surrounded by lymphocytes (star) are observed ( × 400). (d) Liver from rat injected with 200 mg thioacetamide $kg^{-1}$ body wt, seven days. A portal space is observed without alterations (star). The hepatocytes are free of lesions and contain glycogen vacuoles ( × 120).

**Figure 4** (a) Enzymatic activities in plasma of rats injected with DMNA at several doses. *Ordinates*: (left) AST (IU $mg^{-1}$ protein plasma), white bars. ALT (IU $mg^{-1}$ protein plasma), dashed bars. (right) HP activity (UA $mg^{-1}$ protein plasma), solid line. *Abscissas*: DMNA (mg $kg^{-1}$ body wt). (b) Enzymatic activities in plasma of rats injected with DMNA at several doses. *Ordinates*: see **Figure 2(a)**. *Abscissas*: thioacetamide in mg $kg^{-1}$ body wt.

documented in studies of the metabolism of xenobiotics (Alvares, 1982). We present here the mean of three cases processed separately.

The purity of HP preparations was checked by SDS-polyacrylamide gel electrophoresis, producing a single band in every case (data not shown).

### Discussion

The action of hepatotoxic substances on rat liver seems to provoke a regenerative wave when the doses used produce limited necrosis. Thioacetamide, a hepatocarcinogen, stimulates DNA synthesis and mitosis in rats (Morley & Boyer, 1977). Carbon tetrachloride, following toxic liver injury, produces synthesis of DNA in non-necrotic areas (Leevy *et al.*, 1959). In spite of this evidence, the factor or factors responsible for this apparent regenerative process in the wake of a limited necrosis remain unknown.

The goal of this work was twofold: (1) to detect the presence of a liver DNA synthesis promoter, HP, previously isolated by our group (Díaz-Gil *et al.*, 1986), in situations of hepatic aggression, and (2) to determine whether there could be a correlation in time between increased hepatic injury and higher concentration of this HP.

We chose as aggressive agents DMNA and thioacetamide. Both toxins provoke liver necrosis, although they seem to produce, at least in part, differing liver changes at the ultrastructural level (Svoboda *et al.*, 1967).

In spite of these apparently different mechanisms of action, both of them are able to induce the appearance of HP activity in plasma. The possibility of assigning to this HP some function in liver regeneration is very attractive. The results shown in Figures 4A and 4B, where injection of high doses of DMNA or thioacetamide is followed by the detection of greater activity of HP, seem to support this hypothesis. Moreover, the results shown in Figures 1A and 1B seem to suggest the same possibility. Here, the HP activity was only detectable in the 'acute phase' (higher levels of AST and ALT), provoked either by DMNA or thioacetamide, but neither in controls (uninjected rats) nor in rats returned to normal values of AST and ALT, without necrotic areas, was there the slightest evidence of it (Figures 2D and 3D). In the case of thioacetamide-injected rats, AST and ALT concentration was only checked at 0, 48 and 72 h. The apparent maximum (48 h) was chosen by analogy with the DMNA experiments and previous results from other authors (Morley & Boyer, 1977).

The extent of the regenerative wave produced by both toxins, in quantitative terms, is shown in Table I. The possible discrepancy between the HP activity detected in plasma of rats injected with 60 mg DMNA $kg^{-1}$ body wt and absence of mitotic figures in liver, could be interpreted taking into consideration the absence of hepatocytes in the areas affected by the carcinogen. It seems that the action of DMNA has been so intense as to make hepatocytes disappear in these areas. In spite of that, the question could be posed as to why HP does not act on hepatocytes other than those directly affected by DMNA. This finding remains to be explained. One possible explanation is that these high doses of DMNA provoke such changes at the hepatocyte membrane level, so that HP cannot act on its target sites. (When HP from these rats is injected into mice, it produces a stimulation of DNA synthesis in hepatocytes which seems to support this hypothesis.) Another possibility is that in these situations of massive necrosis, HP alone is not sufficient to induce DNA synthesis and that other factors are required.

In this context, some authors have reported the existence of liver growth factors (Morley & Kingdon, 1973; Russell *et al.*, 1984; Nakamura *et al.*, 1984). The possible relationship of any of these factors to the HP purified by our group is unknown at present, and equally unknown is the importance of these growth factors in the regenerative process studied in this work.

This study does not indicate the extent of the necrotic areas (% of necrotic liver or number of necrotic cells per lobule) necessary to proke the appearance of HP activity, or whether there are some specific, more sensitive areas in the liver which induce the appearance of the HP in plasma. Although it could be suggested that the necrosis detected in pericentrilobular (DMNA) or centrilobular (thioacetamide) areas may be responsible for switching on the regenerative wave, these areas are also the prime target of the toxic agents. In any case, there seems to be a clear relationship between the existence of necrosis in the liver and the detection of this HP in plasma, which suggests some role of the HP in the regenerative process. The timing of the appearance of this regeneration factor and its biological importance in the development of the regenerative process remain to be determined.

We have indicated previously that HP preparations apparently show the same molecular weight in partial hepatectomy situations as in cases of DMNA and thioacetamide-injection. As these different preparations show wide variations in activity, we have to conclude that the change of the 'inactive' to the 'active' form of HP could imply minor changes at the molecualr level (amino acid sequence, phosphorylation or methylation of some residues, etc.), not detectable by this technique. We are investigating the alteration(s) that could explain the process of activation of HP.

G.S. is recipient of a fellowship from CAICyT 82/1475/86.

We thank Ms Martha Messman for her help in typing the manuscript and the entire group of the Department of Experimental Biochemistry of the Clínica Puerta de Hierro for their help and useful comments.

This work was supported by grant 83/0629 from the Fondo de Investigaciones Sanitarias (F.I.S.S.), Spain.

### References

ALVARES, A.P. (1982). Oxidative biotransformation of drugs. In *The Liver. Biology and Pathobiology*, Arias, I.M. *et al.* (eds) p. 265. Raven Press: New York.

BURTON, K. (1968). Determination of DNA concentration with diphenylamine. In *Methods in Enzymology*, **12** Colowick, S.P. & Kaplan, N.O. (eds) p. 163. Academic Press: New York and London.

DÍAZ-GIL, J.J., ESCARTIN, P., GARCIA-CAÑERO, R. & 8 others (1986*a*). Purification of a liver DNA synthesis promoter from plasma of partially hepatectomized rats. *Biochem. J.*, **235**, 49.

DÍAZ-GIL, J.J., SANCHEZ, G., SANTAMARIA, L., TRILLA, C., ESTEBAN, P. & ESCARTIN, P. (1986*b*). Liver DNA synthesis promoter activity detected in human plasma from subjects with hepatitis. *Hepatology*, **6**, 658.

HIGGINS, G.M. & ANDERSON, R.M. (1931). Experimental pathology of the liver: Restoration of the liver of the white rat following partial surgical removal. *Arch. Pathol. (Chicago)*, **12**, 186.

IVANETICH, K.M., EIDNE, K.A., ZIMAN, M.R. & KIRSCH, R.E. (1984). Liver injury and regeneration. In *The Liver Annual*, Arias, I.M. *et al.* (eds) p. 97. Elsevier: Amsterdam.

LAEMMLI, U.K. (1980). Cleavage of structural proteins during the assembly of the head of bacteriophage T4. *Nature*, **227**, 680.

LEEVY, C.M., HOLLISTER, R.M., SCHMID, R., MACDONALD, R.A. & DAVIDSON, C.S. (1959). Liver regeneration in experimental carbon tetrachloride intoxication. *Proc. Soc. Exp. Biol. and Med.*, **102**, 672.

LOWRY, O.H., ROSEBROUGH, N.J., FARR, A.L. & RANDALL, R.J. (1951). Protein measurement with the folin phenol reagent. *J. Biol. Chem.*, **193**, 265.

MACMANUS, J.P., FRANKS, D.J., YOUDALE, T. & BRACELAND, B.M. (1972). Increases in rat liver cyclic AMP concentrations prior to the initiation of DNA synthesis following partial hepatectomy or hormone infusion. *Biochem. Biophys. Res. Comm.*, **49**, 1201.

MORLEY, C.G.D. & BOYER, J.L. (1977) Stimulation of hepatocellular proliferation by a serum factor from thioacetamide-treated rats. *Biochem. Biophys. Acta*, **477**, 165.

MORLEY, C.G.D. & KINGDON, H.S. (1973). The regulation of cell growth. I. Identification and partial characterization of a DNA synthesis stimulating factor from the serum of partially hepatectomized rats. *Biochim. Biophys. Acta*, **308**, 260.

NAKAMURA, T., NAWA, K. & ICHIHARA, A. (1984) Partial purification and characterization of hepatocyte growth factor from serum of hepatectomized rats. *Biochem. Biophys. Res. Comm.*, **122**, 1450.

RUSSELL, W.E., MCGOWAN, J.A. & BUCHER, N.L.R. (1984) Partial characterization of a hepatocyte growth factor from rat platelets. *J. Cell. Physiol.*, **119**, 183.

SVOBODA, D., RACELA, A. & HIGGINSON, J. (1967). Variations in ultrastructural nuclear changes in hepatocarcinogenesis. *Biochem. Pharmacol.*, **16**, 651.


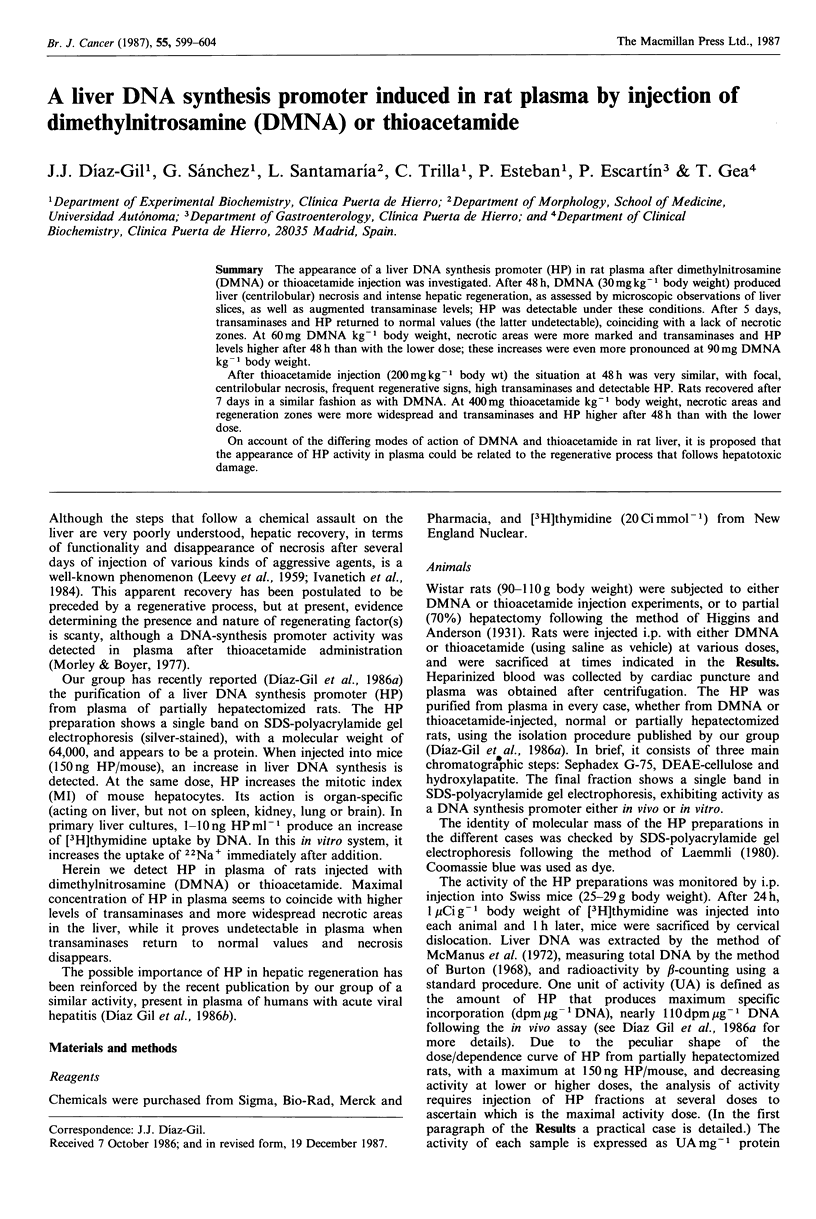

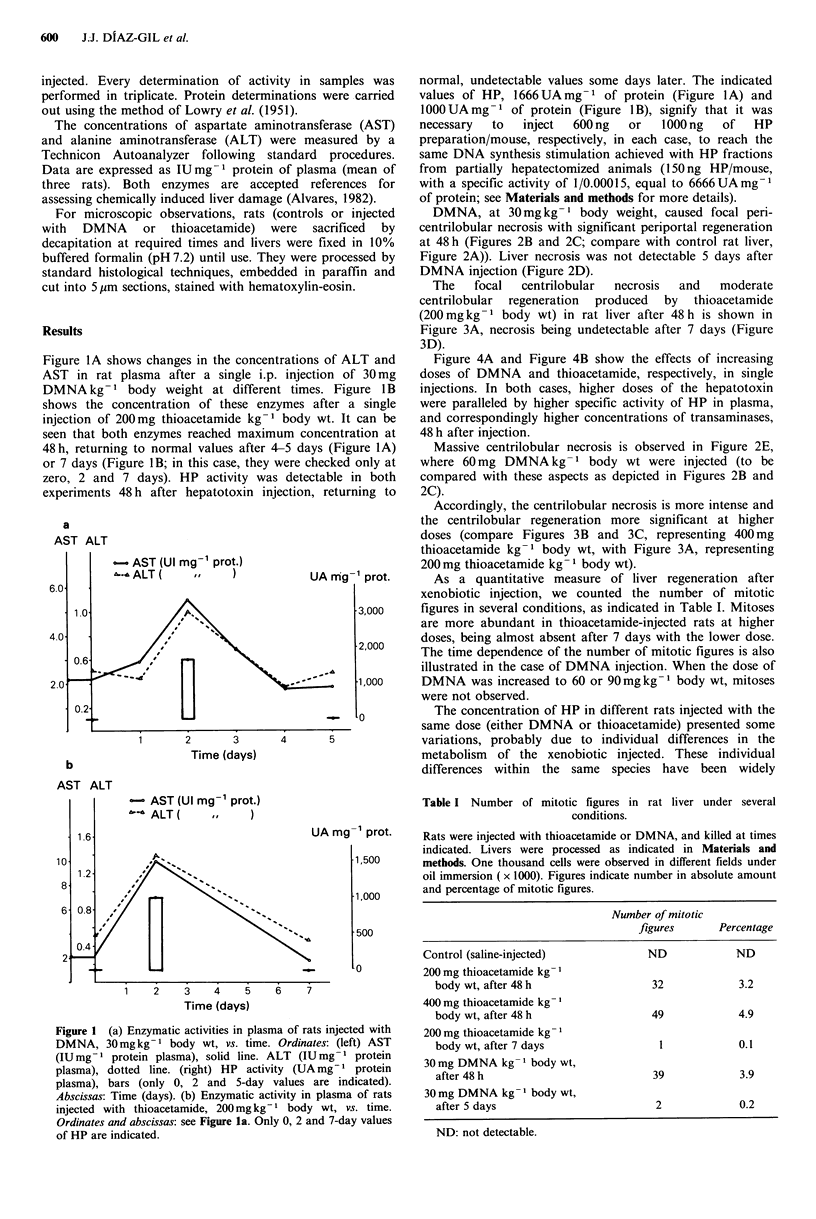

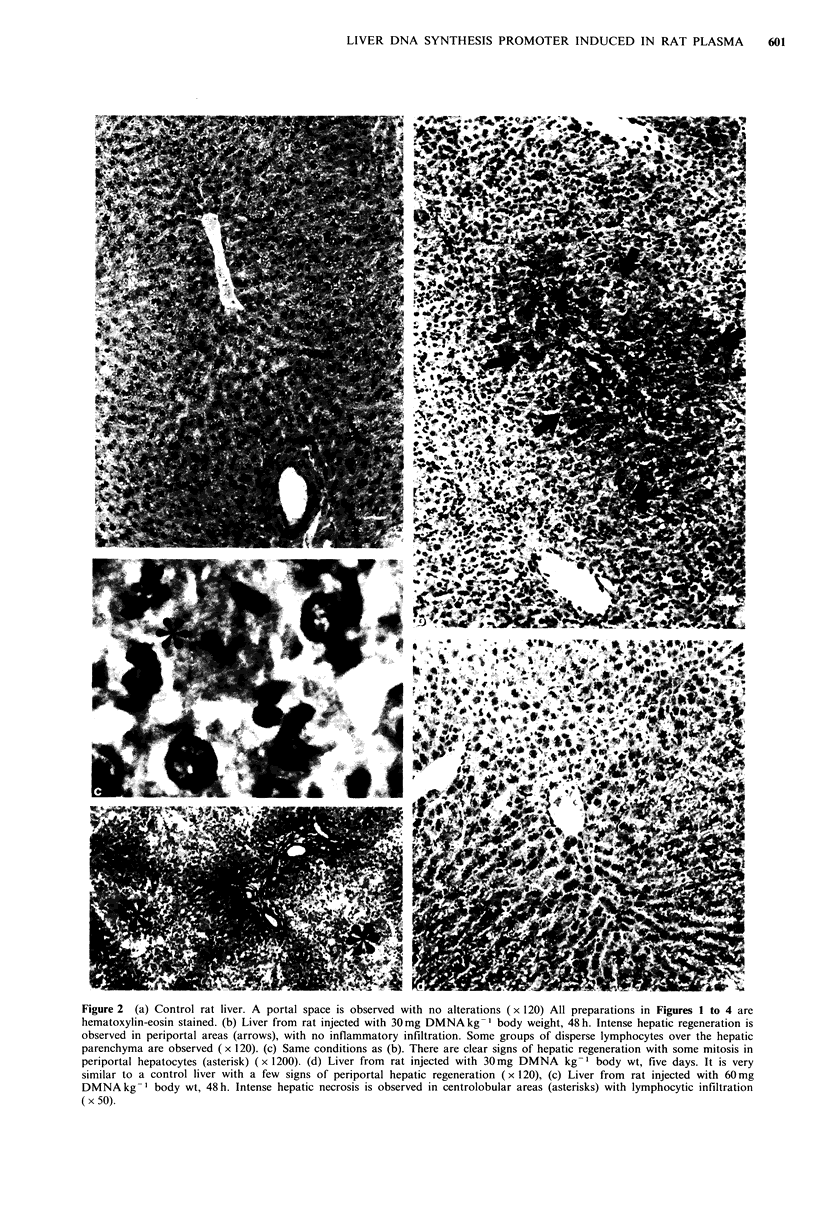

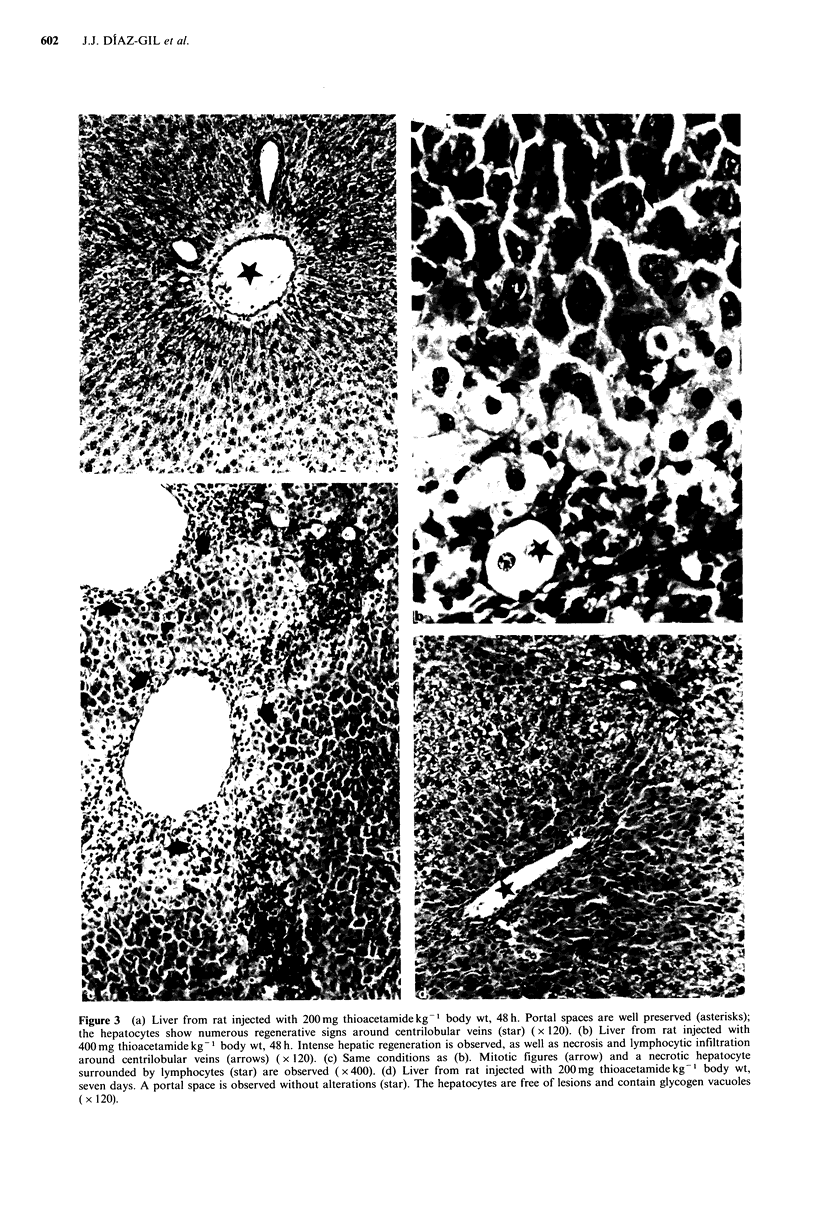

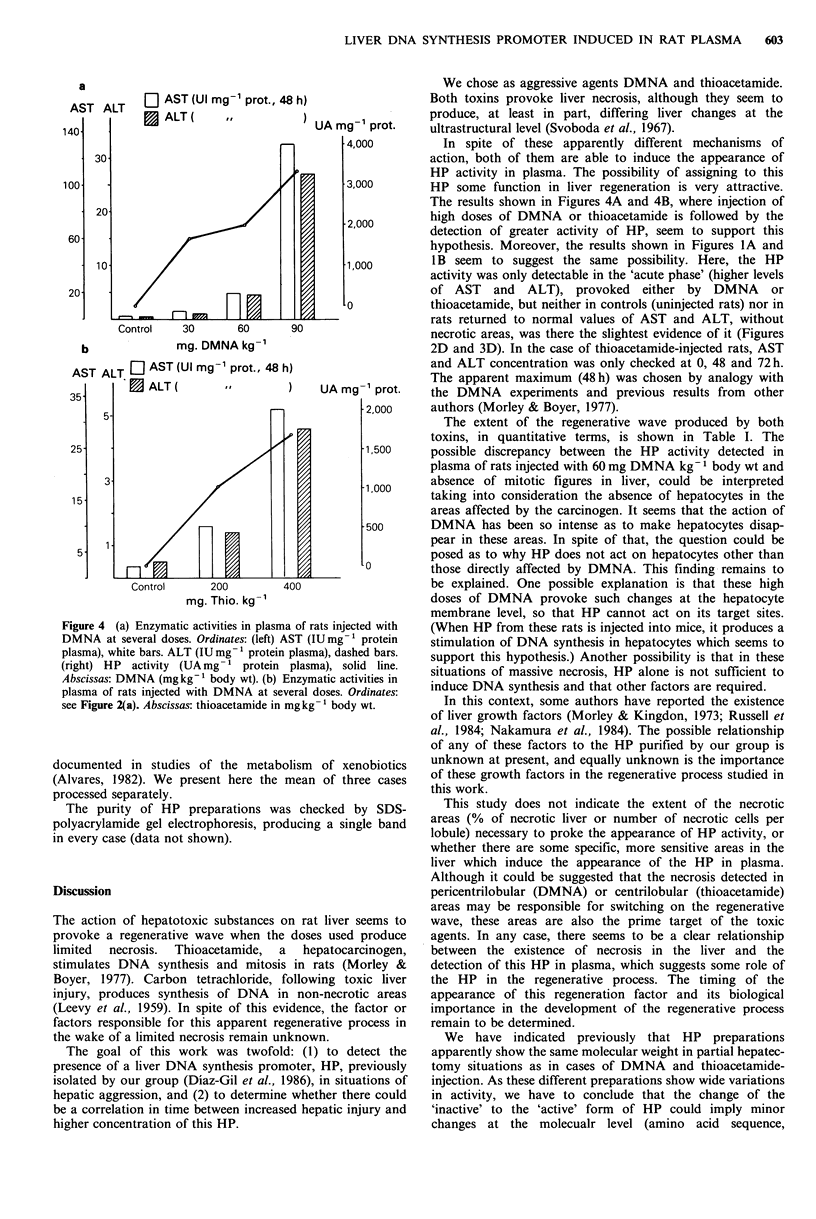

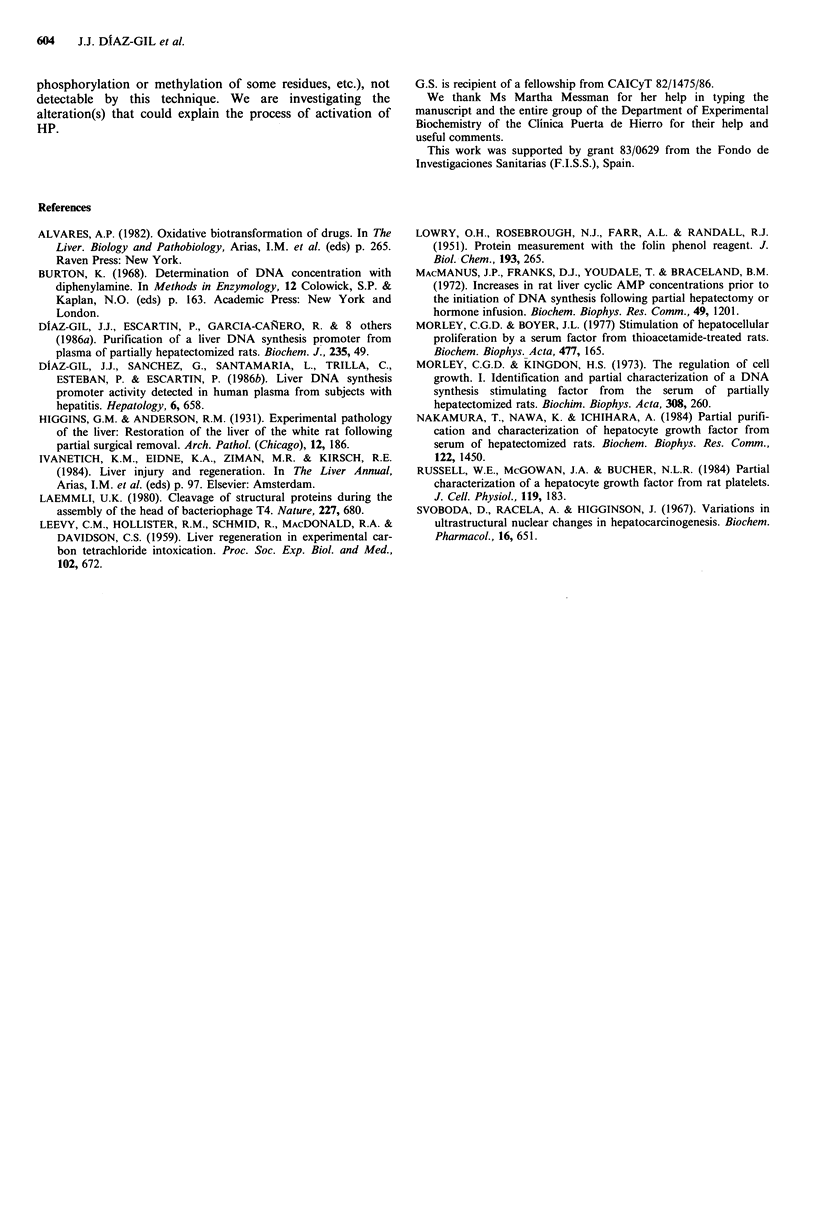


## References

[OCR_00552] Díaz-Gil J. J., Sánchez G., Santamaría L., Trilla C., Esteban P., Escartín P. (1986). Liver DNA synthesis promoter activity detected in human plasma from subjects with hepatitis.. Hepatology.

[OCR_00572] LEEVY C. M., HOLLISTER R. M., SCHMID R., MACDONALD R. A., DAVIDSON C. S. (1959). Liver regeneration in experimental carbon tetrachloride intoxication.. Proc Soc Exp Biol Med.

[OCR_00578] LOWRY O. H., ROSEBROUGH N. J., FARR A. L., RANDALL R. J. (1951). Protein measurement with the Folin phenol reagent.. J Biol Chem.

[OCR_00568] Laemmli U. K. (1970). Cleavage of structural proteins during the assembly of the head of bacteriophage T4.. Nature.

[OCR_00583] Macmanus J. P., Franks D. J., Youdale T., Braceland B. M. (1972). Increases in rat liver cyclic AMP concentrations prior to the initiation of DNA synthesis following partial hepatectomy or hormone infusion.. Biochem Biophys Res Commun.

[OCR_00589] Morley C. G., Boyer J. L. (1977). Stimulation of hepatocellular proliferation by a serum factor from thioacetamide-treated rats.. Biochim Biophys Acta.

[OCR_00594] Morley C. G., Kingdon H. S. (1973). The regulation of cell growth. I. Identification and partial characterization of a DNA synthesis stimulating factor from the serum of partially hepatectomized rats.. Biochim Biophys Acta.

[OCR_00600] Nakamura T., Nawa K., Ichihara A. (1984). Partial purification and characterization of hepatocyte growth factor from serum of hepatectomized rats.. Biochem Biophys Res Commun.

[OCR_00606] Russell W. E., McGowan J. A., Bucher N. L. (1984). Partial characterization of a hepatocyte growth factor from rat platelets.. J Cell Physiol.

[OCR_00611] Svoboda D., Racela A., Higginson J. (1967). Variations in ultrastructural nuclear changes in hepatocarcinogenesis.. Biochem Pharmacol.

